# The effect of long non-coding RNAs in joint destruction of rheumatoid arthritis

**DOI:** 10.3389/fcell.2022.1011371

**Published:** 2022-10-03

**Authors:** Hanxiao Zhao, Li Li, Ning Zhao, Aiping Lu, Cheng Lu, Xiaojuan He

**Affiliations:** ^1^ Institute of Basic Research in Clinical Medicine, China Academy of Chinese Medical Sciences, Beijing, China; ^2^ The Second Clinical College of Guangzhou University of Chinese Medicine, Guangzhou, China; ^3^ Law Sau Fai Institute for Advancing Translational Medicine in Bone and Joint Diseases, School of Chinese Medicine, Hong Kong Baptist University, Kowloon, Hong Kong SAR, China; ^4^ Shanghai GuangHua Hospital of Integrated Traditional Chinese and Western Medicine, Institute of Arthritis Research, Shanghai Academy of Chinese Medical Sciences, Shanghai, China

**Keywords:** rheumatoid arthritis, lncRNAs, joint destruction, cells, ceRNA

## Abstract

Rheumatoid arthritis (RA) is a systemic autoimmune disease accompanied with joint destruction. Serious joint destruction will eventually lead to disability and the decline of life quality in RA patients. At present, the therapeutic effect of drugs to alleviate joint destruction in RA is limited. Recently, accumulating evidences have shown that long non-coding RNAs (lncRNAs) play an important role in the pathogenesis of joint diseases. Therefore, this paper reviews the expression change and the action mechanism of lncRNAs in joint destruction of RA in recent years. A more comprehensive understanding of the role of lncRNAs in joint destruction will help the treatment of RA.

## Introduction

Rheumatoid arthritis (RA) is a disease characterized by progressive symmetrical inflammation and joint destruction. In inflamed joints, chronic destructive synovitis forms, causing irreversible damage to cartilage and bone, which can eventually lead to joint deformities and affect the normal life of RA patients (Weyan and Goronzy. 2021). Up to now, there are limited drugs targeting joint destruction of RA. Therefore, it is necessary to further investigate the molecular mechanism responsible for joint destruction of RA so as to develop novel therapeutic strategies.

In eukaryotic cells, about 90% of RNAs cannot encode proteins, and these RNAs are called non-coding RNAs. In non-coding RNAs, those with a length greater than 200 nucleotides are called long non-coding RNAs (lncRNAs), and those with a length of about 22 nucleotides are called microRNAs (miRNAs) (Deviatkin et al., 2020). LncRNAs can interact with DNA, RNA and protein to regulate transcription, modify epigenetics, maintain the stability, translation and post-translational modification (Bridges et al., 2021).Current studies show that lncRNAs have the following action mode (Bhat et al., 2016; Dykes and Emanueli. 2017): lncRNAs can interact with chromatin-modifying enzymes and RNA-binding factors to activate or inhibit the transcription of target genes. LncRNAs can be an enhancer or a decoy for the transcription factor to promote or repress the transcription. Besides, lncRNAs, as negative regulators, regulate miRNAs expression and function by specifically sponging their corresponding miRNAs, which is called as the competing endogenous RNAs (ceRNAs). This competitive relationship is realized through the combination of miRNA reaction elements (MREs) contained in lncRNAs or mRNAs and miRNAs seed sequences. LncRNAs are now known to participate in many biological processes such as cell proliferation, development and apoptosis, as well as disease progression including cancer, diabetes and cardiovascular disease (He et al., 2017; Simion et al., 2019; Nandwani et al., 2021). Recently, accumulating evidences have indicated that lncRNAs also play an important role in the pathogenesis of RA (Lodde et al., 2020; Huang et al., 2021). They can regulate the development and function of a variety of cells related to RA, including fibroblast-like synoviocytes (FLSs), osteoclasts, osteoblasts, and chondrocytes, etc. (Zhu et al., 2019; Guo et al., 2020; Li, et al., 2020; Aurilia et al., 2021), hence influencing the progression of joint destruction.

Therefore, this paper aimed to summarize the action mechanism of lncRNAs in various cells of RA joints and their important effect on joint destruction in recent years. The potential of lncRNAs as promising drug targets in joint destruction of RA was also discussed. We searched the original English articles from 2017 to 2022 through PubMed with the following keywords: rheumatoid arthritis and lncRNAs and joint destruction, rheumatoid arthritis and lncRNAs and T lymphocytes, rheumatoid arthritis and lncRNAs and B lymphocytes, rheumatoid arthritis and lncRNAs and macrophages, rheumatoid arthritis and lncRNAs and fibroblast-like synoviocytes, rheumatoid arthritis and lncRNAs and chondrocytes, rheumatoid arthritis and lncRNAs and osteoblasts, rheumatoid arthritis and lncRNAs and osteoclasts, rheumatoid arthritis and lncRNAs and treatment. Comments and articles in other languages were excluded. We hope this review will provide clues for further study of lncRNAs as biomarkers and effective therapeutic targets in treating joint destruction of RA.

## Joint destruction in RA

Joints are composed of osteoclasts, osteoblasts, chondrocytes, synovial cells and other types of immune cells such as T lymphocytes and B lymphocytes. These cells interact with each other and jointly maintain joint homeostasis.

In the maintenance of joint homeostasis, the balance of bone homeostasis is very important. In healthy joints, bone formation promoted by osteoblasts and bone resorption mediated by osteoclasts jointly maintain the dynamic balance of bone tissue. However, in the joints of RA, this balanced relationship is broken, resulting in bone destruction (Zhao et al., 2020). Mature osteoclasts are formed from expressing CD14^+^ and CD16^−^ circulating monocytes/macrophages and tissue-specific macrophages after fusion and multinucleation (Rana et al., 2018; Yao et al., 2021). The combination of receptor activator of nuclear factor κB ligand (RANKL) and macrophage colony-stimulating factor (M-CSF) with their respective ligands activates downstream related signaling pathways affecting the formation and development of osteoclasts (Chen et al., 2018; Győri and Mocsal. 2020). The inflammatory environment of RA joint promotes osteoblasts and other cells to secrete RANKL, thereby promoting osteoclast-mediated bone resorption (Auréal et al., 2020). Osteoblasts are differentiated from mesenchymal stem cells by activating various cell growth factors, transcription factors, and multiple signaling pathways such as wingless/integrated (WNT), bone morphogenetic protein (BMP) signaling pathways (Neve et al., 2011). In the past, osteoclasts obtained intensive investigations in the field of bone destruction research in RA. In contrast, osteoblasts have received less attention. Recently, studies have reported that the maturation and mineralization of osteoblasts was compromised in RA, and regulating the signaling pathways of osteoblasts could effectively attenuate bone destruction and simultaneously promote bone formation in RA (Berardi et al., 2021).

Chondrocytes are the only cell in cartilage, which can produce and maintain cartilage matrix. In RA, the function of chondrocytes is dysregulated. On the one hand, cytokines such as tumor necrosis factor-alpha (TNF-α) and interferon-γ (IFN-γ) can interfere with chondrogenesis and promote chondrocyte apoptosis (Tseng et al., 2020). On the other hand, matrix metalloproteinases (MMPs) secreted by chondrocytes themselves can also accelerate the destruction of cartilage (Otero and Goldring. 2007).

Besides the osteoclasts, osteoblasts and chondrocytes, there are also other types of cells directly or indirectly involved in the pathological process of joint destruction in RA. FLSs, as the main non immune cells in synovial tissue, are generally believed to play central roles in RA progression. They can promote joint destruction in a variety of ways. FLSs participate in osteoclastogenesis by secreting RANKL and M-CSF, and they also have the ability to directly migrate and even invade articular cartilage (Harre and Schett. 2017; Yoshitomi 2019; Nygaard and Firestein. 2020). In addition, FLSs can accelerate cartilage destruction by secreting MMPs, such as MMP-3 and MMP-9. Clinical studies have shown that the level of MMP-3 in serum can effectively reflect the activity of RA disease and joint and bone injury (Lerner et al., 2018). MMP-9 is also proved to be closely related to bone resorption of RA (Cabral-Pacheco et al., 2020).

In RA, T lymphocytes infiltrate into the synovial membrane where they can initiate and inhibit the activities of other cells. T lymphocytes of subtypes such as helper T (Th)1 and Th17 cells can up-regulate the expression of receptor activator of nuclear factor- κB (RANK) in osteoclast precursors. However, some subtypes of T cells can inhibit the development and maturation of osteoclast precursors by secreting IL-4 (Tanaka 2019; Deng et al., 2021). For osteoblasts, T lymphocytes can also regulate the differentiation of osteoblasts in both directions (Deng et al., 2021).

The role of B lymphocytes in joint destruction of RA has been emphasized in recent years. B lymphocytes can inhibit the differentiation of osteoblasts by producing TNF-α and C-C motif chemokine 3 (CCL3) (Sun et al., 2018).They can also produce RANKL to promote the differentiation of osteoclast precursors (Meednu et al., 2016). In addition, autoantibodies such as rheumatoid factor (RF) produced by B lymphocytes also participate in the pathogenesis of RA joint destruction. Harre U and their colleagues found that the immune complex formed by RF and autoantigens can induce osteoclasts differentiation through Fcγ receptors (FcγR) in RA (Harre et al., 2015).

## LncRNAs in joint destruction of RA

### LncRNAs and FLSs

FLSs are crucial participants in the joint destruction of RA. In FLSs of RA, the expression of a few of lncRNAs were abnormal. LncRNA PICSAR, lncRNA LINK-A and lncRNA ZFAS1 expression were up-regulated (Bi et al., 2019; Wang, et al., 2021; Wang, et al., 2021).While lncRNA THRIL expression was down-regulated (Zou et al., 2021). Inhibition of lncRNA PICSAR, lncRNA LINK-A and lncRNA ZFAS1 expression could weakened the invasion ability of FLSs, whereas lncRNA THRIL knockout showed the opposite results.

Besides influencing the invasion of FLSs, lncRNAs also modulate the production of MMPs in FLSs of RA. Research results explained that lncRNA NEAT1 promoted the secretion of MMP-9 in FLSs of RA *via* interacting with miR-410-3p and miR-204-5p (Wang et al., 2020; Xiao et al., 2021). Another study showed that silence of lncRNA THRIL inhibited MMP-3 production in FLSs of RA by regulating the phosphatidylinositol 3-kinases (PI3K)/protein kinase B (AKT) signaling pathway (Liang et al., 2020). One study indicated that overexpression of lncRNA OSER1-AS1 could decreased the production of MMP-3 in FLSs of RA. The mechanism was that lncRNA OSER1-AS1 served as a ceRNA through the sponge of miR-1298-5p and increased the expression of early two factor transcription factor 1 (E2F1) (Fu et al., 2022). In addition, another study found lncRNAS56464. One could bind to miR-152-3p, and then affect the expression of WNT1 in FLSs of RA (Jiang et al., 2021). As a secretory glycoprotein, WNT1 is a core member of WNT signaling pathway, which has been proved to be closely related to the progression and pathogenesis of RA by regulating articular chondrogenesis and bone destruction (Malysheva et al., 2016).

### LncRNAs and osteoclasts

Osteoclasts are the major players directly responsible for the pathogenesis of joint destruction in RA. Numerous research have proved that the proliferation and differentiation of osteoclasts were promoted, leading to the enhancement of bone resorption in RA (Tateiwa et al., 2019; Kitaura et al., 2020).

One study reported that the expression of lncRNA X inactive specific transcript (Xist) was significantly increased in female patients with RA. LncRNA Xist could promote the proliferation of osteoclasts, and this promotion effect could be enhanced after overexpression of lncRNA Xist (Bost et al., 2022). Further, several researchers investigated the mechanism of lncRNA Xist in regulating the proliferation and development of osteoclasts. Shao et al. demonstrated that lncRNA Xist contributed to osteoclast differentiation through serving as a ceRNA of miR-590-3p to promote Tgif2 level (Shao et al., 2021). Other findings suggested that lncRNA Xist promoted osteoclast differentiation through sphingosine kinase 1 (SPHK1)/sphingosine 1-phosphate (S1P)/extracellular signal-regulated kinase (ERK) signaling pathway. It could interact with fused in sarcoma (FUS) and increase the stability of SPHK1 (Zhang et al., 2022a). Although these articles reported the mechanism of action of lncRNA Xist in osteoclasts from different aspects, the role of lncRNA Xist in RA joint destruction still needs to be further verified by *in vivo* experimental data.

Besides lncRNA Xist, some other lncRNAs have been also proved to have the effect on regulating osteoclastogenesis. A study confirmed that lncRNA SNHG15 was highly expressed in THP-1 cells stimulated by M-CSF and RANKL. Overexpression of lncRNA SNHG15 could promote the proliferation, differentiation and metastasis of osteoclasts through sponging with miR-381–3p to upregulate the expression of never-in-mitosis-A-related kinase 2 (NEK2) (Wang, et al., 2022). Another research indicated that lncRNA NEAT1 also played an important role in osteoclast differentiation. Knockdown of lncRNA NEAT1 impaired osteoclastogenesis, whereas overexpression of lncRNA NEAT1 promoted osteoclastogenesis. Mechanistically, lncRNA NEAT1 sponged with miR-7 and blocked miR-7-mediated regulation of protein tyrosine kinase 2 (PTK2). Moreover, this study showed that rs12789028 could act as a strong allele-specific functional enhancer for lncRNA NEAT1 (Zhang et al., 2020b).

Recently, with the advances in RNA sequencing (RNA-seq)) technology, lncRNA-mRNA expression profiles have become the research hotspot. A couple of lncRNA-mRNA co-expression network was established and analyzed. Through RNA-seq analysis, a previous study noted that a lot of lncRNAs and mRNAs differentially expressed in human osteoclast differentiation. Then, researchers constructed a lncRNA-mRNA co-expression network and found that lncRNA ENSG00000257764.2 obtained the highest number of interactions and interacted with tissue inhibitor of metalloproteinases 2 (TIMP2), which was an inhibitor of MMPs (Li, et al., 2020). Hypoxia-inducible factor 1α (HIF-1α) has been implicated in the pathogenesis of RA and it participates in osteoclast differentiation by regulating nutrient and energy sensors (Fearon et al., 2016; Tang et al., 2019). Therefore, another study explored the lncRNA-mRNA expression profiles associated with HIF-1α-knockout mouse osteoclast differentiation by RNA-seq (Tian et al., 2022). The results showed that lncRNA MSTRG.31769.2 and MSTRG.7566.12 were the two lncRNAs with the two highest numbers of interactions. The expression of these two lncRNAs had a strong negative correlation with MMP-9 and cathepsin K (CTSK) expression. However, most of these results are from *in vitro* experiments and lack of further validation in RA animal models and patients.

### LncRNAs and osteoblasts

Albeit osteoblasts are among the dominant cell types in the joint, their role in joint destruction of RA has only been received attention in recent years. Interference of the growth and differentiation of osteoblasts is one of the important factors causing joint destruction of RA.

The expression of lncRNA Colorectal Neoplasia Differentially Expressed (CRNDE) in various cancer tissues and plasma is sensitive and specific (Lu et al., 2020). Recent studies have demonstrated that lncRNA CRNDE could promote the proliferation and differentiation of osteoblasts through WNT signaling pathway (Maeda et al., 2019). Mice with lncRNA CRNDE knockout showed reduced bone mass. Moreover, the proliferation and differentiation of osteoblasts isolated from lncRNA CRNDE knockout mice were inhibited (Mulati et al., 2020). LncRNA differentiation antagonizing non-protein coding RNA (DANCR) play an important role in many diseases. A study demonstrated that lncRNA DANCR suppressed the differentiation of mesenchymal stem cells by promoting the degradation of Skp2-induced forkhead box O1 (FOXO1) ubiquitination. Silencing lncRNA DANCR could promote the maturation and mineralization of osteoblasts (Tang et al., 2018). Although the expression level of lncRNA DANCR was up-regulated in the bone tissue of patients undergoing total hip replacement, this expression change has not been confirmed in patients or animal models with RA.

In addition to lncRNA CRNDE and lncRNA DANCR, increasing *in vitro* cell experiments showed the effects of lncRNAs in regulating the proliferation, differentiation and mineralization of osteoblasts. By sponging miR-33a, lncRNA MCF2L-AS1 positively regulated the expression of Runx2 and promoted the differentiation and calcium nodule formation in human bone marrow mesenchymal stem cells (BMSCs) (Chen, Wang, et al., 2020). Linc-ROR could promote osteogenesis of mesenchymal stem cells by functioning as a ceRNA for miR-138 and miR-145. The ALP activity and calcium nodules formation were increased in linc-ROR-overexpressing cells but decreased in linc-ROR-knockdown cells (Feng et al., 2018). Nevertheless, despite some reports of lncRNAs and osteoblasts, the function and mechanism of these lncRNAs in joint destruction of RA are still largely unknown, which deserve further studies.

### LncRNAs and chondrocytes

As an important part of the joint, chondrocytes also contribute to the progressive destructive process of RA. Chondrocytes are the target cells of multiple inflammatory factors. Simultaneously, chondrocytes also act as effector cells, directly or indirectly facilitate joint damage of RA (Tseng et al., 2020).

As a member of a family of isozymes, pyruvate dehydrogenase kinase 4 (PDK4) is one of the most important factors which direct carbon flux into glycolysis from oxidative phosphorylation (Li, et al., 2020). It can promote proliferation and migration of chondrocytes through the RANKL/RANK/osteoprotegerin (OPG) pathway. Recent studies indicated that PDK4 was decreased in chondrocytes of RA (Liu, et al., 2020). The results of one study pointed out that lncRNA GAS5 was overexpressed in LPS-induced chondrocytes. It decreased the proliferation of chondrocytes by sponging miR-361–5p to modulate PDK4 expression (Zhang et al., 2021). Another research reported that knockout of lncRNA GAS5 could prevented cartilage destruction and reduce the expression of MMP-13 and FGF21 in cartilage of antigen-induced arthritis (AIA) mice (Chen, et al., 2020). Besides, Gao et al. showed that lncRNA GAS5 could induce apoptosis of chondrocytes from osteoarthritis patients through down-regulating miR-137 (Gao et al., 2020). Although lncRNA GAS was confirmed to be upregulated in cartilage and serum of patients with osteoarthritis, there is still a lack of validation in serum and cartilage of patients with RA.

Simultaneously, the function of several other lncRNAs in chondrocytes have also been investigated by *in vitro* experiments. LncRNA MEG3 and lncRNA ZNF667-AS1 were down-regulated in LPS-stimulated chondrocytes. These two lncRNAs could protect chondrocytes from LPS-induced damage and inhibit the secretion of inflammatory factors by chondrocytes (Li et al., 2019; Zhuo et al., 2021).

### LncRNAs and T lymphocytes

In normal environment, a variety of cytokines secreted by T cells maintain a dynamic balance in the proliferation, development, differentiation and apoptosis of osteoblasts, osteoclasts and chondrocytes. Many lncRNAs were found to be abnormal in T cells of patients with RA. LncRNA GAS5, lncRNA RMRP, lncRNA THRIL and lncRNA NEAT1 were found to be increased in T cells of RA patients (Moharamoghli et al., 2019; Shui et al., 2019; Liu et al., 2021).

As major subsets of CD4^+^ Th cells, Th17 cells not only promoted joint inflammation, but also induced osteoclasts differentiation and bone destruction of RA. IL-17 secreted by Th17 cells can promote the production of RANKL by osteoblasts and synoviocytes, thus indirectly inducing osteoclasts differentiation (Hashimoto 2017; Yang et al., 2019; Tang et al., 2020). One study pointed out that the expression of lncRNA NEAT1 was up-regulated in Th17 cells differentiated by CD4+T cells of RA *in vitro* (Shui et al., 2019). Another study suggested that lncRNA NEAT1 in serum derived exosomes could promote the proliferation of CD4+T cells and differentiate into Th17 cells (Liu et al., 2021). Through exosomes, circulating lncRNAs can be delivered to recipient cells and participate in the pathogenesis of RA.

### LncRNAs and B lymphocytes

In RA, a variety of cytokines and autoantibodies secreted by B cells can inhibit the differentiation of osteoblasts and chondrocytes, as well as promote the formation of osteoclasts. Although the role of B cells in the progression of RA disease has been extensive investigated, there are few studies on lncRNAs and B cells in RA. It is well known that glucocorticoids inhibit the survival of osteoblasts and promote the life span of osteoclasts through glucocorticoid receptors, leading to the risk of bone loss (Güler-Yüksel et al., 2018; Lee et al., 2021).One study found that the expression of lncRNA GAS5 in B cells of RA patients was obviously decreased. The pathogenesis of lncRNA GAS5 involved in RA may be related to the inhibition of glucocorticoid receptors via its decoy RNA “glucocorticoid response element” (Mayama et al., 2016).

### LncRNAs’ multiple effects

Interestingly, not only different lncRNAs can regulate the same cell, a lncRNA can also act on different cells and perform different functions as well. For instance, one study showed that lncRNA GAS5 accelerated cartilage destruction by regulating PI3K/AKT/fibroblast growth factor 21 (FGF21) axis through sponging miR-103 (Chen et al., 2020). Other studies pointed that lncRNA GAS5 promoted apoptosis of FLSs and suppress their proliferation by promoting histone deacetylase 4 (HDAC4) and sirtuin 1 (Sirt1) via inhibiting miR-128–3p and miR-222–3p (Peng et al., 2021; Yang et al., 2021). In addition, one study showed that through binding miR-181c-5p, lncRNA SNHG1 induced BMSCs differentiation into osteoclasts, whereas inhibited the osteogenic differentiation of BMSCs (Yu et al., 2021). Other studies reported that lncRNA MEG3 could promote the proliferation and differentiation of osteoblasts by activating WNT signaling pathway, and affect the differentiation of macrophages into osteoclasts by inhibiting interferon regulatory factor 8 (IRF8) (Li et al., 2019; Gao et al., 2022). The above results indicate that the functions of lncRNAs are variety and complex.

## Treatment

Given that lncRNAs participate in the development of joint destruction, focusing on lncRNAs could be a promising strategy for treatment of RA. Bioactive glass nanoparticles (BGN) are attractive for orthopedic applications (Kong et al., 2018). A study reported that BGN induced BMSCs to secrete extracellular vesicles containing lncRNA NRON, which could prevent osteoclasts differentiation by blocking the nuclear translocation of nuclear factor of activated T cell cytoplasmic 1 (NFATc1), a core member of the nuclear factor of activated T cell (NFAT) family of transcription factors that involved in osteoclasts differentiation (Gu et al., 2020; Yang et al., 2022). This study provides a hopeful strategy for the treatment of bone-related disease including RA. Besides that, some drugs for RA have also been found to regulate the expression of lncRNAs involved in joint destruction. Methotrexate is the guideline recommended first-line treatment for RA (Smolen et al., 2020). Iguratimod is a new drug that can promote bone formation and inhibit bone resorption (Xie et al., 2020).Combined treatment with methotrexate and iguratimod reduced the expression of lncRNA HOTAIR in RA patients (Tan et al., 2021). Recently, research has reported that lncRNA HOTAIR could promote osteoclasts differentiation, inhibit osteoclasts apoptosis and osteoblasts differentiation (Shen et al., 2019; Dong et al., 2022).

In addition to being a drug target, lncRNAs can also be used as a biomarker to judge the efficacy of drugs. Etanercept, the first TNF inhibitor used in the treatment of RA, can effectively improve bone loss and inhibit joint destruction in RA patients (Gulyás et al., 2020).Some researchers reported that after etanercept treatment in RA patients, there were significant differences in the expression of some lncRNAs between the responders and non-responders. These lncRNAs were enriched into osteoclasts differentiation signaling pathways, etc. (Wang et al., 2022). Tripterysium Glycosides Tablets (TGT), derived from Tripterygium wilfordii Hook F, have been used to treat RA in clinic for many years. TGT can effectively improve the tissue architecture of joints and prevent the bone destruction in RA (Wang et al., 2017). However, clinical evidence shows that only 70% of RA patients respond to TGT. Recently, researchers found a TGT response-related lncRNA. Their study showed that the expression of lncRNA ENST000000494760 in the effective group of TGT was significantly down-regulated compared with that in the ineffective group. Further studies indicated that overexpressing lncRNA ENST00000494760 promoted C1qC expression by sponging with miR-654–5p (Zhang, Wang, et al., 2020). C1qC is one of polypeptide chains of C1q. A study showed that C1q could enhance osteoclasts development (Teo et al., 2012).

Collectively, these studies indicated that lncRNAs might provide targets and evaluation indicators for personalized drug therapy of joint destruction in RA.

## Conclusion and prospect

At present, the treatment of bone destruction in RA is still a big challenge in clinic. The research progress of lncRNAs in bone destruction of RA provides us with a new therapeutic idea. The role of lncRNAs in RA bone destruction is complex and diverse. LncRNAs can regulate various type of cells in joints of RA through different ways, ultimately leading to joint destruction. However, there are still some limitations in the current research on lncRNAs’ role in bone destruction of RA. On the one hand, majority of studies about lncRNAs in RA joint destruction are limited to cellular levels, lacking further verification from *in vivo* experiments and clinical trials. On the other hand, the role of lncRNAs in regulating some important cells such as macrophages in the joint of RA are still unclear. This might be one of the next research directions of lncRNAs in RA. Furthermore, current studies on lncRNAs in joint destruction mostly focus on evaluating the change of expression levels and exploring the mechanisms of action, with less experimental data on pathological implications as well as diagnosis and treatment potential, which deserve deeper research in future. Additionally, owing to the complex and diverse role of lncRNAs in different pathways and different cells, some new technologies such as multi-omics and systemic biology are best introduced in in-depth studies.

**FIGURE 1:**
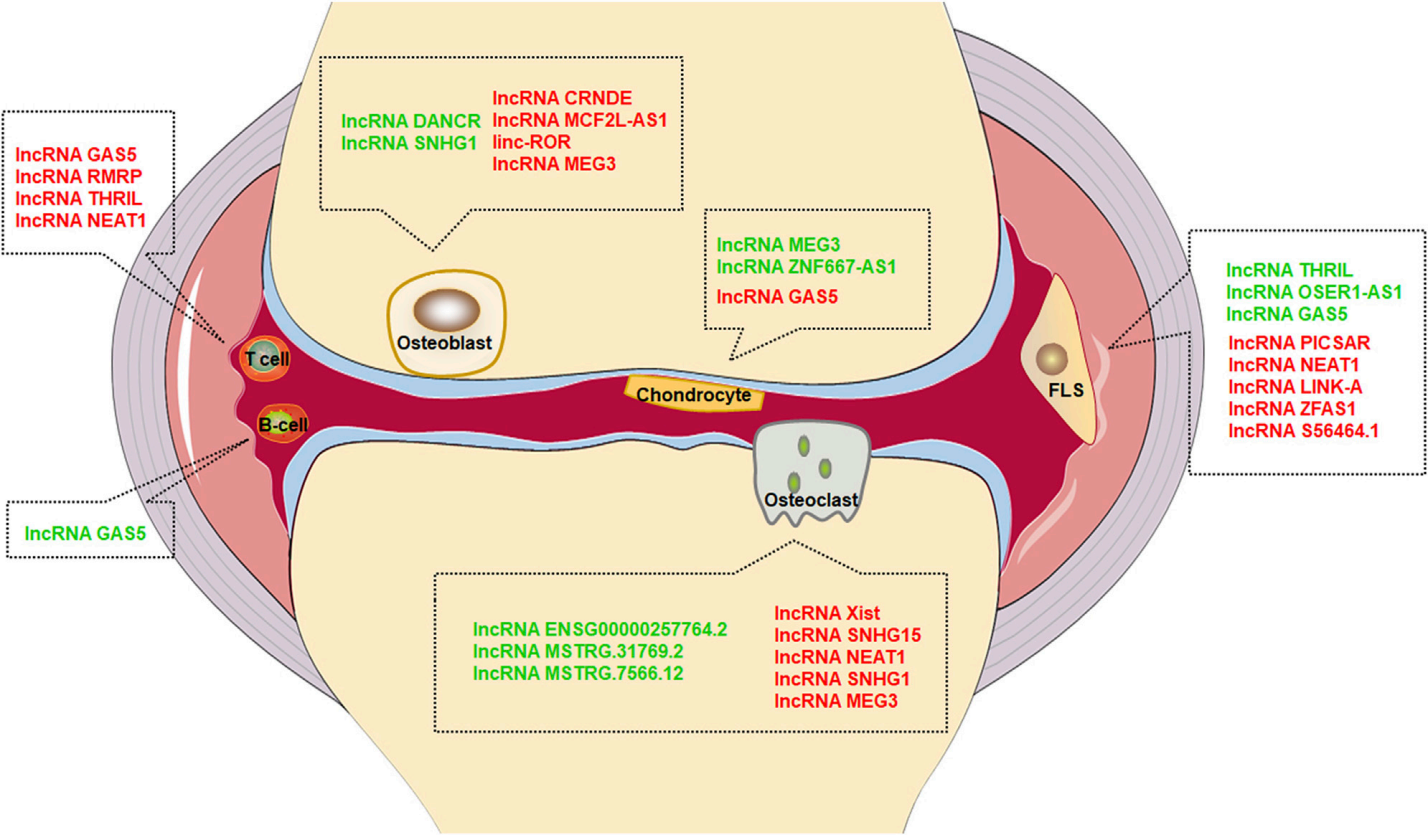
Effect of lncRNAs in different cells. Green font represents inhibition, red font represents promotion.

To sum up, we reviewed the potential and action mechanism of lncRNAs participating in joint destruction and suggested that joint destruction of RA can be alleviated by regulating lncRNAs (Table 1 and Figure 1). LncRNAs can serve as potential biomarker and therapeutic target for RA joint destruction.

**TABLE 1:** Roles of lncRNAs in joint destruction of RA.

lncRNAs	Model	Targets	Signaling	Roles	References
lncRNA PICSAR	FLSs	miR-4701–5p	—	promote FLSs proliferation, migration and invasion	Bi et al. (2019)
lncRNA LINK-A	FLSs	miR-1262	—	promote FLSs invasion	Wang et al. (2021)
lncRNA ZFAS1	FLSs	miR-3926	—	promote FLSs proliferation, migration, invasion	Wang et al. (2021)
lncRNA NEAT1	FLSs	miR-410–3p; miR-204–5p	—	promote FLSs migration, invasion, and inflammatory cytokines secretion	Wang et al. (2020); Xiao et al. (2021)
lncRNA S56464.1	FLSs	miR-152–3p	WNT signaling pathway	promote FLSs proliferation	Jiang et al. (2021)
lncRNA THRIL	FLSs	MMP3	PI3K/AKT signaling pathway	inhibit FLSs proliferation, migration, invasion	Liang et al. (2020); Zou et al. (2021)
lncRNA OSER1-AS1	FLSs	miR-1298–5p	—	inhibit FLSs propagation, release of inflammatory factor	Fu et al. (2022)
lncRNA GAS5	FLSs	miR-128–3p; miR-222–3p	—	inhibit FLSs proliferation	Peng et al. (2021); Yang et al. (2021)
lncRNA Xist	osteoclasts from RA women; RAW264.7 cells; BMMs	miR-590–3p; FUS	SPHK1/S1P/ERK signaling pathway	promote the proliferation and differentiation of osteoclasts	Shao et al. (2021); Bost et al. (2022); Zhang et al. (2022)
lncRNA SNHG15	THP-1 cells	miR-381–3p	—	promote the proliferation, differentiation and metastasis of osteoclasts	Wang et al. (2022)
lncRNA NEAT1	BMMs and PBMCs	miR-7	—	promote osteoclast differentiation	Zhang et al., 2022
lncRNA ENSG00000257764.2	CD14 monocytes	TIMP2	—	—	Liu et al. (2020)
lncRNA MSTRG.31769.2	RAW264.7 cells	—	—	—	Tian et al. (2022)
lncRNA MSTRG.7566.12	RAW264.7 cells	—	—	—	Tian et al. (2022)
lncRNA MEG3	RAW264.7 cells	IRF8	—	affect osteoclasts differentiation	Gao et al. (2022)
lncRNA SNHG1	BMSCs	miR-181c-5p	WNT signaling pathway	inhibit osteoclast differentiation and induce osteoblast differentiation	Yu et al. (2021)
lncRNA CRNDE	MC3T3-E1 cells and CRNDE knockout mice	—	WNT signaling pathway	promote osteoblast proliferation and differentiation	Mulati et al. (2020)
lncRNA MCF2L-AS1	BMSCs	miR-33a	—	promote osteogenic differentiation	Chen et al. (2020)
linc-ROR	BMSCs	miR-138; miR-145	WNT signaling pathway	promote osteogenic differentiation	Feng et al. (2018)
lncRNA DANCR	BMSCs	FOXO1	—	inhibit osteoblast differentiation	Tang et al. (2018)
lncRNA MEG3	MC3T3-E1 cells	—	WNT signaling pathway	promote proliferation and differentiation of osteoblasts	Li, et al. (2019)
lncRNA GAS5	chondrocytes	miR-361–5p; miR-137	—	involve in chondrocytes proliferation and apoptosis	Gao et al. (2020); Zhang et al. (2021)
lncRNA MEG3	chondrocytes	miR-141	AKT/mTOR signaling pathway	promote chondrocytes proliferation	Li et al. (2019)
lncRNA ZNF667-AS1	chondrocytes	miR-523–3p	JAK/STAT signaling pathway	promote chondrocytes proliferation	Zhuo et al. (2021)
lncRNA GAS5	T lymphocytes	—	—	—	Moharamoghli et al. (2019)
lncRNA THRIL	T lymphocytes	—	—	—	Moharamoghli et al. (2019)
lncRNA RMRP	T lymphocytes	—	—	—	Moharamoghli et al. (2019)
lncRNA NEAT1	T lymphocytes	STAT3; miR-144–3p	WNT signaling pathway	promote CD4+T cells to differentiate into Th17 cells	Shui et al. (2019); Liu et al. (2021)
lncRNA GAS5	B lymphocytes	—	—	—	Mayama et al. (2016)
